# Current state of and need for enzyme engineering of 2-deoxy-D-ribose 5-phosphate aldolases and its impact

**DOI:** 10.1007/s00253-021-11462-0

**Published:** 2021-08-19

**Authors:** Juha Rouvinen, Martina Andberg, Johan Pääkkönen, Nina Hakulinen, Anu Koivula

**Affiliations:** 1 Department of Chemistry, University of Eastern Finland, PO Box 111, FI-80101 Joensuu, Finland; 2 VTT Technical Research Centre of Finland Ltd, P.O. Box 1000, FI-02044 VTT Espoo, Finland

**Keywords:** DERA, Aldolase, Protein engineering, C–C bond formation, Biocatalysis, Applications

## Abstract

**Abstract:**

Deoxyribose-5-phosphate aldolases (DERAs, EC 4.1.2.4) are acetaldehyde-dependent, Class I aldolases catalyzing in nature a reversible aldol reaction between an acetaldehyde donor (C2 compound) and glyceraldehyde-3-phosphate acceptor (C3 compound, C3P) to generate deoxyribose-5-phosphate (C5 compound, DR5P). DERA enzymes have been found to accept also other types of aldehydes as their donor, and in particular as acceptor molecules. Consequently, DERA enzymes can be applied in C–C bond formation reactions to produce novel compounds, thus offering a versatile biocatalytic alternative for synthesis. DERA enzymes, found in all kingdoms of life, share a common TIM barrel fold despite the low overall sequence identity. The catalytic mechanism is well-studied and involves formation of a covalent enzyme-substrate intermediate. A number of protein engineering studies to optimize substrate specificity, enzyme efficiency, and stability of DERA aldolases have been published. These have employed various engineering strategies including structure-based design, directed evolution, and recently also machine learning–guided protein engineering. For application purposes, enzyme immobilization and usage of whole cell catalysis are preferred methods as they improve the overall performance of the biocatalytic processes, including often also the stability of the enzyme. Besides single-step enzymatic reactions, DERA aldolases have also been applied in multi-enzyme cascade reactions both in vitro and in vivo. The DERA-based applications range from synthesis of commodity chemicals and flavours to more complicated and high-value pharmaceutical compounds.

**Key points:**

• *DERA aldolases are versatile biocatalysts able to make new C–C bonds.*

*• Synthetic utility of DERAs has been improved by protein engineering approaches.*

*• Computational methods are expected to speed up the future DERA engineering efforts.*

**Graphical abstract:**

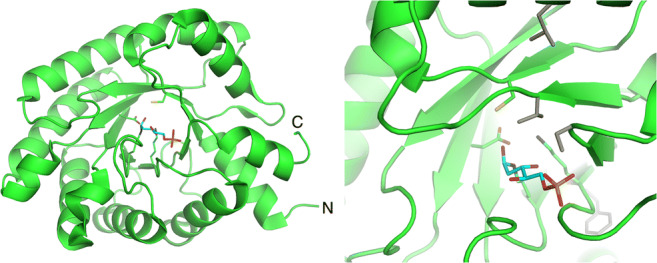

## Introduction

Enzymatic C–C bond formation reactions offer a means to build up the carbon backbone and synthesize important organic compounds. The C–C bond forming reactions can be catalyzed by enzymes belonging to various EC classes, such as lyases, oxidoreductases, and transferases. The most well-known and studied reactions are catalyzed by aldolases (EC 4.1.2, belonging to lyases), transketolases (EC 2.2.1, belonging to transferases), and pyruvate decarboxylases (EC 4.1.1.1, belonging to lyases). The latter two enzyme families utilize thiamine diphosphate as a cofactor. In addition, decarboxylation renders to overall reaction irreversible that might be useful in increasing the reaction yield (Resch et al. 2011; Fesko and Gruber-Khadjawi 2013). The use of various aldolases in stereoselective synthesis and their engineering has been recently reviewed (Clapés et al. 2010; Windle et al. 2014). The current review focuses in particular on 2-deoxyribose-5-phosphate aldolases (DERA, EC 4.1.2.4), which are widespread cytosolic enzymes involved in the catabolism of the pentose motif of deoxynucleosides in the pentose phosphate pathway and do not require any cofactor for their activity (Tozzi et al. 2006).

**Fig. 1:**
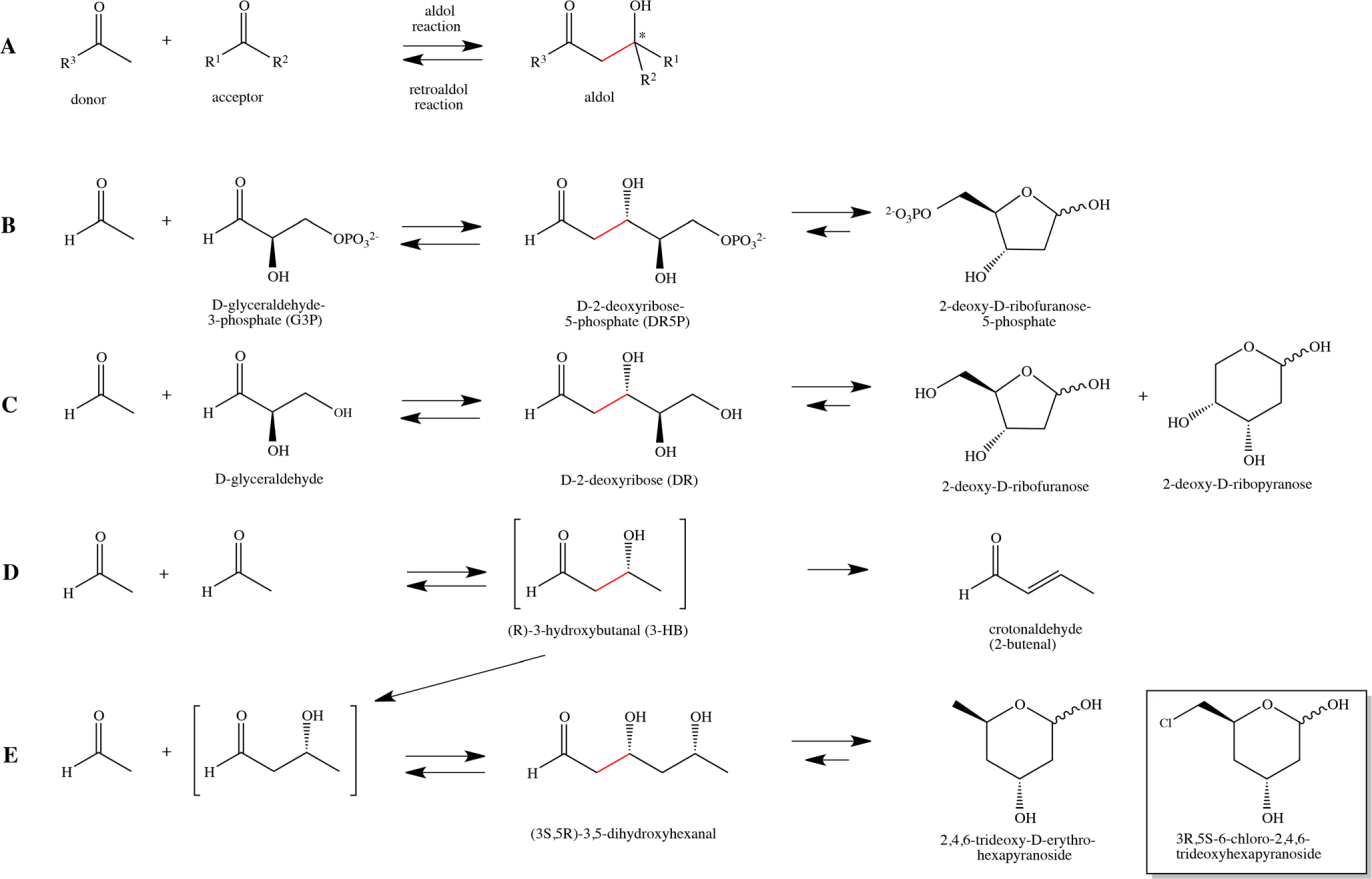
Chemical reactions catalyzed by DERA. (A) A general aldol reaction in which the carbonyl compound donor makes a covalent bond (in red) with the carbonyl compound acceptor. Depending on the donor, there is a possibility for formation of a new stereogenic center (*) in the product. (B) A reversible aldol reaction catalyzed in Nature by DERA, a Class I aldolase, between an acetaldehyde donor (C2 compound) and glyceraldehyde-3-phosphate acceptor (C3 compound, G3P) to generate deoxyribose-5-phosphate, DR5P (C5 compound) which forms a furanose isomer. (C) DERA-catalyzed reaction between acetaldehyde and a non-phosphorylated glyceraldehyde produces D-2-deoxyribose (DR), which is able to form furanose and pyranose isomers. (D) The reaction of two acetaldehydes, catalyzed by DERA, produces crotonaldehyde. (E) Crotonaldehyde can react further with a third acetaldehyde molecule in a DERA-catalyzed reaction, to produce 3,5-dihydroxyhexanal, which cyclizes to a more stable pyranose form

**Fig. 2:**
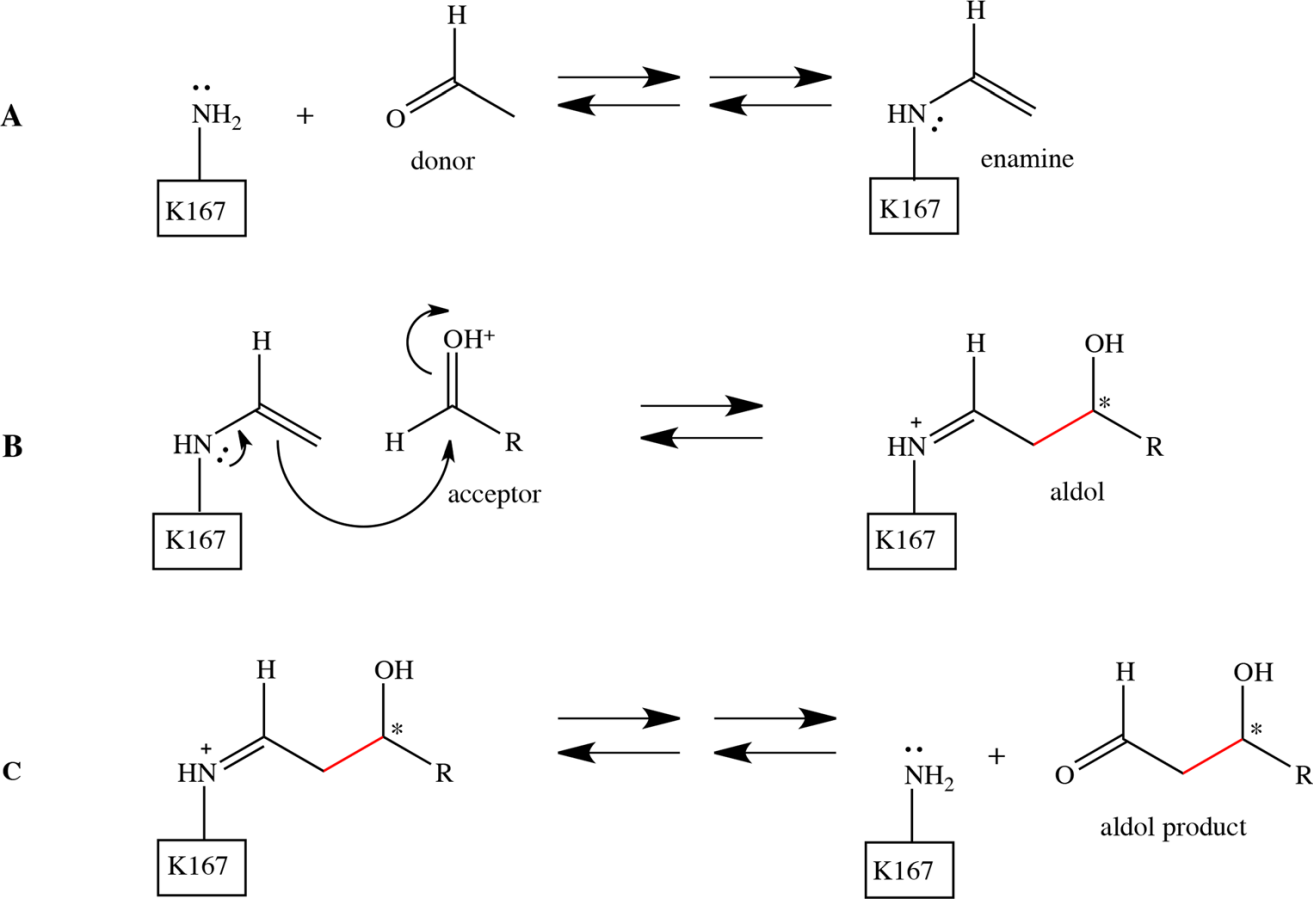
Main phases in the reaction mechanism of DERA-catalyzed aldol reaction. (A) The donor aldehyde makes a covalent bond with nucleophilic Lysine (Lys167 in *E. coli* DERA) of the enzyme, forming an enamine (in equilibrium with imine, the Schiff base). This enamine forming phase includes several intermediates. (B) Reaction of the enamine with the protonated acceptor aldehyde produces an aldol, covalently attached to the Lysine. (C) Release of aldol. This phase also includes several intermediates. The key catalytic residue is Lysine which make a covalent bond with the donor aldehyde. In addition, there are other residues in the active site which promote the reaction participating in protonation and deprotonation of intermediates

Aldolases, in general, catalyze the reversible formation of C–C bonds by the aldol addition of a nucleophilic donor, for example a ketone enolate, onto an electrophilic aldehyde acceptor (Fig. 1A). DERA enzymes belong to so called Class I aldolases and catalyze a reaction for which both of the substrates and the product are aldehydes, i.e. the reaction between 2-deoxy-D-ribose-5-phosphate (DR5P) and D-glyceraldehyde-3-phosphate (G3P) + acetaldehyde (C2) (Fig. 1B). In this reversible reaction, C–C bond is either cleaved (retroaldol reaction) or formed (aldol reaction) (Fig. 1A). Class I aldolases form with the donor substrate a Schiff base (an imine), which is in equilibrium with enamine form and able to react with the acceptor aldehyde (Fig. 2).

DERA enzymes are promiscuous and able to accept a wide range of non-natural aldehydes as acceptor molecules, thus offering a biocatalytic alternative for a (stereo)selective synthesis of C–C bonds, as shown for example by Liu and Wong (2002). Donor substrate specificity is stricter; however, there are studies demonstrating that some DERA enzymes also possess nucleophile substrate promiscuity (Barbas et al. 1990; Hernández et al. 2018; Chambre et al. 2019). Furthermore, DERA enzymes can use acetaldehyde as sole substrate, leading in a tandem reaction to formation of a C6 product, 2,4,6-trideoxyhexose (Fig. 1D–E), which is cyclized spontaneously and can be removed from the reaction (Gijsen and Wong 1994). In an aldol addition step, catalyzed by DERA, a new stereogenic center is formed (Fig. 1A). It should also be noted that reaction products of DERA are also aldehydes, and these can be readily subjected to further addition reactions leading to increasingly complex structures. DERA enzymes have been used to synthesize commodity chemicals like pentaerythritol (a building block, e.g. for the synthesis of explosives, plastics, paints, and cosmetics), deoxysugars, flavours, and complex pharmaceutical molecules. The latter include antitumor agent epothilone A, and investigational Islatravir drug for HIV treatment. DERA-based processes have also been developed for the synthesis of side chain of statin-type cholesterol-lowering drugs (i.e. atorvastatin and rosuvastatin) by DSM Pharma Chemicals, Diversa and Lek Pharmaceuticals. The biocatalytic applications of DERA have been recently extensively reviewed (Bolt et al. 2008; Fei et al. 2017; Haridas et al. 2018)

The use of DERA enzymes would thus offer great potential in manufacturing various chemicals (Fei et al. 2017; Haridas et al. 2018). One of the major challenges in using naturally occurring DERA enzymes in industrial processes, in which high substrate loads are needed to reach the desired productivity, is the high inactivation of the enzyme in the presence of aldehydes. In addition, the catalytic efficiency on non-natural substrates is often considerably lower as compared to the natural substrates. Furthermore, if the acceptor and donor substrate ranges could be further broadened or shifted, this would also expand the application potential of DERA aldolases. Protein engineering offers a means to affect the enzyme properties, such as substrate promiscuity and catalytic rate, to optimize or invert stereoselectivity, and to improve stability under certain conditions or release inhibition. Recent developments in this field concerning DERA aldolases are the scope of this review.

## Sequence and structural information of DERA enzymes

DERA aldolases are ubiquitous enzymes found in all kingdoms of life including fungi, bacteria, archaea, and higher eukaryotes, as well as psychrophilic and hyperthermophilic organisms. As explained in more details below, DERA enzymes share a common TIM barrel fold. In a phylogenetic analysis of approximately 2500 DERA sequences, obtained from the KEGG Orthology database using the KO identifier for *Escherichia coli* DERA, Kim et al. found that DERA enzymes are clustered into five major groups (Kim et al. 2020). The different groups included sequences from bacteria, Firmicutes (*Bacilli* and *Clostridia*), Proteobacteria (including *E. coli* DERA), and two groups with sequences from mixed domains.

The 3D structures of several DERA enzymes have been solved, and there are also some complex structures available. These have revealed that despite relatively low sequence identity, all DERAs have a TIM (α/β)_8_-barrel fold where the catalytic amino acids as well as other amino acid residues around the active site seem to be relatively well conserved (Heine 2001; Haridas et al. 2018). DERA enzymes usually have a quaternary structure, which in the reported cases is either a dimer or tetramer (Dick et al. 2016b). DERA aldolases derived from psychrophilic and mesophilic organisms seem to have dimeric structure, whereas those from hyperthermophilic have tetrameric structure. Moreover, the dimer interface for psyhcrophilic and mesophilic DERAs seem similar. Hydrophobic clusters within the dimer interfaces are suggested to contribute to the overall thermostability of tetrameric DERAs from thermophilic bacterium *Thermus thermophilus* and hyperthermophilic archaeon *Aeropyrum pernix* (Sakuraba et al. 2003; Lokanath et al. 2004). In general, the thermostabilities of DERA enzymes seem to vary for reasons that are not self-evident. As an example, DERA derived from *E. coli*, a mesophilic bacterium, is relatively thermostable, having the measured melting temperature (*T*_m_) of 65 °C (Voutilainen et al. 2020).

**Fig. 3:**
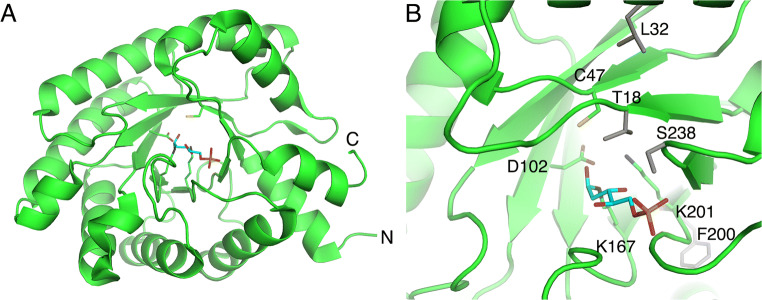
(A) The 3D structure of monomeric DERA from *E. coli*. (B) The active site of *E. coli* DERA with the covalently bound carbinolamine intermediate (in cyan) (PDB 1JCL). Key catalytic residues are presented as green sticks. The positions of four mutations described in the review are presented as grey sticks

The first solved crystal structure of DERA was that from *E. coli* (Heine 2001; Heine et al. 2004). It is also the most well-studied and applied DERA enzyme so far. The polypeptide chain of *E. coli* DERA consists of 259 amino acid residues; however, the crystal structure did not contain the last eight residues at C-terminus, probably due to existence of multiple conformations. It has been shown by NMR spectroscopy that this tail exists indeed in two major conformations and the C-terminal Tyrosine participates also in catalysis (Schulte et al. 2017; Schulte et al. 2018). The *E. coli* DERA crystal structure showed the formation of a symmetric dimer. The moderate monomer-monomer interface (444 Å^2^), however, suggests that enzyme exists as a mixture of monomer and dimer in solution. The monomer of DERA contains one eight-stranded α/β-barrel and the active site is located at the C-terminal region of the parallel β-sheet barrel. According to the complex structures and mutagenesis studies, it was deduced that Lys167 is a key catalytic residue making a covalent bond with donor aldehyde (Heine 2001). Asp102 and Lys201 are also important for the reaction course participating in protonation and deprotonation of reaction intermediates (Heine 2001). Cys47 seems not to be essential for catalysis but its reaction with reaction products or intermediates may lead to enzyme inactivation (Heine 2001; Dick et al. 2016a; Bramski et al. 2017) (Fig. 3).

In the substrate-binding pocket of DERA, the donor acetaldehyde binds to the bottom and the acceptor G3P (glyceraldehyde-3-phosphate) to the upper part of the pocket. Binding of the substrates is mediated through hydrogen bonds, either directly or through water molecules. The substrate-binding site of DERA is highly preferential for the phosphorylated acceptor substrate, G3P, which is illustrated by the drastic loss of activity when comparing G3P and D-glyceraldehyde as acceptor substrates (Table 1). The main residues for phosphate binding in *E. coli* DERA are Lys172 (via a bridging water molecule), Ser238, and Gly205, and water bridge interactions with the backbone moieties of Gly171, Val206, Gly236, and Ser239.

**Table 1:** Reported kinetics for retroaldol reactions by wild-type DERA from different organisms (the sources which contained *k*_cat_/*K*_m_ values were selected)

Organism	Substrate	*K*_m_ [mM]	*k*_cat_ [1/s]	*k*_*c*at_/*K*_m_ [1/(s•mM)]	Reference
*E. coli*	DR5P	0.64	68	106	Heine (2001)
*E. coli*	DR5P	0.64	68	106	DeSantis et al. (2003)
*E. coli*	DR5P	0.29	40	135	Kullartz and Pietruszka (2012)
*E. coli*	DR5P	0.29	33	115	Bisterfeld et al. (2016)
*R. erythropolis*	DR5P	4.84	18	4	Kullartz and Pietruszka (2012)
*B. halodurans*	DR5P	0.22	13	60	Kim et al. (2020)
*P. atrosepticum*	DR5P	0.22	8,5	39	Haridas et al. (2020)
*E. coli*	DR	57	0,1	0.002	DeSantis et al. (2003)
*E. coli*	DR	54	0,2	0.004	Kullartz and Pietruszka (2012)
*R. erythropolis*	DR	418	1,2	0.003	Kullartz and Pietruszka (2012)

The determination of 3D structures of DERA initiated protein engineering studies in order to improve catalytic efficiency and to develop DERA-based variants for catalysis of aldol reactions for various substrates.

## Kinetic properties and substrate promiscuity of DERA wild-type enzymes

DERA is currently the only member of the acetaldehyde-dependent Class I aldolases, and more generally, it is one of the only two known aldolases, fructose-6-phosphate aldolase being the other, that are able to catalyze aldol reactions between two aldehydes (Garrabou et al. 2009; Chambre et al. 2019). The Class I aldolases do not require any cofactor (unlike Class II aldolases) but exhibit a conserved Lysine residue in the active site. In *E. coli*, DERA-catalyzed reaction acetaldehyde (donor substrate) forms a covalent Schiff base intermediate with the active site Lys167 (Heine 2001), and its enamine tautomer corresponds to the enolic donor in aldol reaction (Fig. 2A). The electrophilic acceptor substrate of DERA is G3P. The product from the DERA-catalyzed aldol addition reaction, DR5P, can exist as linear aldehyde isomer and as cyclic furanose isomer in solution. The covalent bond formation with Lys167 of *E. coli* DERA is only possible with linear isomer of DR5P. On the other hand, the chemical equilibrium of the reaction favours the formation of DR5P in solution because of the stability of cyclic furanose form of DR5P over the linear form (Fig 1B). The equilibrium constant *K* of this aldol reaction (Fig. 1B) has been reported to be 5000 M^−1^ for *Lactobacillus plantarum* DERA (Pricer and Horecker 1960).

Detailed kinetic data have been reported only for a limited number of DERA-catalyzed reactions and include mainly data for retroaldol reaction in which DR5P or DR (D-2-deoxyribose) has been used as a substrate (Fig. 1B–C) (Table 1). DERA is an efficient enzyme when DR5P is used as a substrate although the conversion from cyclic furanose form to linear aldehyde form may affect the kinetics. The catalytic efficiency (*k*_cat_/*K*_m_) for DR is significantly lower due to the weaker substrate binding, which can be seen in much higher (roughly 100-fold in the case of *E. coli* DERA) *K*_m_ values as compared to that of DR5P (Table 1). DR does not have the phosphate group which seems, thus, to be important for tighter binding. As can be seen from Table 1, the turnover number (*k*_cat_) is also much smaller for DR.

DERA aldolases were discovered in 1950s (Racker 1952), and soon the potential of DERA as a tool for enzyme-mediated C–C bond formation between acetaldehyde and various non-natural acceptor substrates became evident (Rosen et al. 1965). Barbas and coworkers (Barbas et al. 1990) showed that with regard to the specificity of acceptors, many aldehydes as well as aldose sugars (e.g. glucose, arabinose, ribose, and N-acetylglucosamine) and their phosphates are accepted as weak substrates. The catalytic efficiency was shown in each case to be up to several orders of magnitude lower towards these types of non-natural acceptor substrates (Chen et al. 1992). Furthermore, it was demonstrated that besides acetaldehyde, DERA can accept propionaldehyde as well as also two ketones, acetone and fluoroacetone, as weak donor substrates. A recent screening of over 300 different DERA sequences, selected from the UniProt database and heterologously expressed in *E. coli*, further revealed that some microbial DERA enzymes can indeed display also remarkable donor substrate promiscuity (Hernández et al. 2018; Chambre et al. 2019). As mentioned earlier, DERA enzymes are additionally able to catalyze tandem aldol addition of acetaldehyde molecules, in which the aldehyde product from the first aldol addition is used as acceptor substrate in the second reaction (Gijsen and Wong 1994). The coupling of three acetaldehydes produces a stable cyclic hexapyranose (Fig. 1D–E), which offers routes for stereospecific manufacturing of cyclic compounds. Sakuraba and coworkers (Sakuraba et al. 2007) have demonstrated that hyperthermophilic DERAs from *P. aerophilum* and *T. maritima* are much more efficient in the production of cyclic hexapyranose compared to *E. coli* DERA, but interestingly in the synthesis of DRP, *E. coli* DERA was more efficient. In addition, Greenberg et al. (2004) screened successfully from large metagenomic libraries DERA enzymes that have a better tolerance to high substrate concentrations and allow reduced usage of catalyst in production of 6-carbon precursor of statin drugs.

## Protein engineering of DERA

Protein engineering to optimize the enzyme properties, such as substrate specificity or activity and/or stability under operational conditions, has classically relied either on rational design, based on solved 3D structures (or good 3D models) of the enzymes, or on using directed evolution without the necessity of any structural information. Directed evolution iteratively evolves an enzyme towards the defined goal by creating large genetic variation to the starting wild-type enzyme sequence (Stemmer 1994; Arnold 2018). Different mutagenesis approaches can be utilized to create these enzyme variant libraries: random mutagenesis, saturation mutagenesis, and/or DNA shuffling (through recombination). Selection, or more often due to practical reasons, screening of variant libraries is used as a means to identify improved enzyme candidates, and usually further rounds of sequence diversification are needed to achieve the desired enzyme properties. Screening can be carried out in various manners using either the desired target substrates or model substrates, such as fluorogenic substrates to aid the assaying (Jourdain et al. 1998; Greenberg et al. 2004; Fei et al. 2015).

Directed evolution is a powerful way to optimize the enzyme properties towards application requirements. However, as the screening, even if carried out in an automated high-throughput manner, may be very time-consuming and costly, computational methods and/or structural information of the enzymes are being more and more incorporated into directed evolution approaches to create targeted (focused) enzyme variant libraries (Powell et al. 2001; Reetz 2013; Yang et al. 2014; Arnold 2018).

DERA properties, which have been engineered, include substrate specificity or preference, decreased inhibition by acetaldehyde, and altered stereoselectivity. We give examples below on these rational design and directed evolution efforts that have been used (see also Table 2). A separate section is also dedicated to machine learning methods utilized to engineer DERA. Most of the DERA-based applications utilize one-step enzymatic reaction, but there are also examples where DERA-catalyzed reaction is a part of multi-enzyme reaction cascade.

**Table 2:** Examples of protein engineering work carried out with DERA aldolases

Protein engineering technology	Reaction of interest	Source of the DERA gene	Substrate	Mutation(s) introduced	Result (as compared to the wild-type DERA)	Reference
Structure-based design	Retroaldol	*E. coli*	DR	S238D	Improved catalytic efficacy and specificity	DeSantis et al. (2003)
Structure-based design	Aldol	*K. pneumoniae*	Acetaldehyde, D-glyceraldehyde	S238D/F200I/∆Y259	Improved catalytic efficacy and aldehyde tolerance	Li et al. (2015)
Structure-based design	Retroaldol	*E. coli*	DR5P	C47L	Reduction of intermediate inhibition	Bramski et al. (2017)
Structure-based design	Aldol	*B. halodurans*	Acetaldehyde	F160Y	Improved catalytic efficiency in aldol reaction	Kim et al. (2020)
Directed evolution	Aldol	*E. coli*	Acetaldehyde, chloroacetaldehyde	F200I	Improved catalytic efficacy	Jennewein et al. (2006)
Directed evolution	Aldol	*E. coli*	Acetaldehyde, chloroacetaldehyde	F200I/ΔY259	Improved catalytic efficacy at high chloroacetaldehyde concentration	Jennewein et al. (2006)
Directed evolution	Aldol	*E. coli*	Acetaldehyde, chloroacetaldehyde	F200I/S258T/Y259T + C-terminal extension KTQLSCTKW	Improved catalytic efficacy at high chloroacetaldehyde concentration	Jennewein et al. (2006)
Homologous grafting and utilization of saturation mutagenesis	Aldol	*E. coli*	Acetaldehyde, propanal	T18S	Altered stereoselectivity	Bisterfeld et al. (2016)
Random mutagenesis	Aldol	*L. brevis*	Acetaldehyde, chloroacetaldehyde	T29L	Improved catalytic efficacy	Jiao et al. (2017)
Directed evolution	Retroaldol	*S. halifaxensis*	Acetaldehyde	S2C/Q10R/ C47V/ D66L/A71V/A145K/ L156I/M184I/ V203I/S235T/S236D	Improved acetaldehyde tolerance	Huffman et al. (2019)
Site-directed mutagenesis and machine learning	Aldol	*E. coli*	Acetaldehyde	C47V/G204A/S239D	Improved catalytic efficacy in aldol reaction, and clearly reduced retroaldol activity on DR5P and DR	Voutilainen et al. (2020)
Site-directed mutagenesis and machine learning	Aldol	*E. coli*	Acetaldehyde	N21S/C47V/G204A	Improved catalytic efficacy in aldol reaction, and clearly reduced retroaldol activity on DR5P and DR	Voutilainen et al. (2020)
Site-directed mutagenesis and machine learning	Aldol	*E. coli*	Acetaldehyde	C47V/G204A/S239E	Improved catalytic efficacy in aldol reaction, and clearly reduced retroaldol activity on DR5P and DR	Voutilainen et al. (2020)
Site-directed mutagenesis and machine learning	Aldol	*E. coli*	Acetaldehyde	C47V/G204A	Improved catalytic efficacy in aldol reaction, and clearly reduced retroaldol activity on DR5P and DR	Voutilainen et al. (2020)
Site-directed mutagenesis and machine learning	Aldol	*E. coli*	Acetaldehyde	N21K/C47V/G204A	Improved catalytic efficacy in aldol reaction, and clearly reduced retroaldol activity on DR5P and DR	Voutilainen et al. (2020)

## Structure-based design

By using the crystal structure of *E. coli* DERA, DeSantis et al. designed five single mutations in the active site to improve retroaldol reaction with DR, the non-phosphorylated derivative of natural substrate, DR5P (DeSantis et al. 2003). In the *E. coli* DERA complex crystal structure, Ser238 forms a hydrogen bond with phosphate of the bound DR5P (Fig. 3B). In line with the structural data, the Ser238Asp mutation showed 2.5-fold improvement in retroaldol reaction with DR when compared to the wild-type enzyme. The mutated enzyme was also having clearly (over 100-fold) reduced activity (*k*_cat_) on DR5P. Interestingly, the mutant enzyme was more potent compared to the wild-type enzyme for using 3-azidopropinaldehyde as an acceptor in a sequential aldol reaction to form a deoxy-azidoethyl pyranose, which can be used in synthesis of cholesterol-lowering drugs such as atorvastatin (Liu et al. 2004). The importance of Ser239 for the binding of the phosphate group of DR5P has also been demonstrated with the Ser239Pro mutation resulting in clearly reduced *K*_m_ value while not affecting the catalytic rate (*k*_cat_) of *E. coli* DERA enzyme (Ma et al. 2016).

It is also worth noting that in the above-mentioned work by DeSantis et al., in vivo selection system to improve the substrate specificity of DERA was utilized (DeSantis et al. 2003). The chosen selection strategy coupled the enzyme activity to growth, which is a very efficient selection principle allowing large amounts of enzyme variants (up to 10^10^–10^13^) to be examined (Boersma et al. 2007). In the study, an engineered *E. coli* SELECT strain requiring acetaldehyde for growth was utilized. The *E. coli* SELECT strain transformed with a plasmid encoding for wild-type DERA enzyme was able to grow when the cells were cultivated on minimal medium supplemented with 2-deoxy-D-ribose 5-phosphate (DR5P), but not if the medium was supplemented with the non-phosphorylated substrate D-2-deoxyribose (DR). On the other hand, if *E. coli* SELECT cells were transformed with DERA variants exhibiting high retroaldol activity on DR (thus providing acetaldehyde needed for growth), the positive transformants would grow in medium supplied with only DR as a substrate (DeSantis et al. 2003). This latter system would thus allow discovery of completely new DERA variants with improved activity on non-phosphorylated or other non-natural substrates.

Li and coworkers have engineered *Klebsiella pneumoniae* DERA for synthesis of 2-deoxycarbohydrates (Li et al. 2015). They used 3D structural model of the enzyme and information from the literature to create a number of variants. The goal was to increase affinity towards non-phosphorylated substrates and tolerance to higher aldehyde substrate concentrations. Aldose reaction by using acetaldehyde and D-glyceraldehyde to produce DR was used to characterize the mutants. The variant KDERA^K12^ (i.e. *K. pneumoniae* DERA having mutations Ser238Asp/Phe200Ile/ΔTyr259) showed 3-fold improvement in specific enzyme activity in this reaction as compared to the DERA wild-type and 4-fold improvement as compared to the *E. coli* DERA. The substrate tolerance towards aldehyde substrates also increased by 1.5-fold. The authors discuss how to rationalize these results. The Ser238 residue forms a hydrogen bond with phosphate group and its mutation modifies substrate specificity towards non-phosphorylated substrates (Fig. 3B). Phe200 is located in the β-strand in the active site but not directly interacting with the substrate (Fig. 3B). Its modification may slightly alter the shape of the active site. Tyr259 is located at C-terminus of the protein and recently it has been reported that this tyrosine could actually enter to the active site in a closed state conformation of DERA (Schulte et al. 2018).

Despite the fact that DERA enzymes utilize acetaldehyde as a substrate, acetaldehyde is known to be inhibitory at high concentrations required in applications. Dick and coworkers have investigated the mechanism of deactivation and suggested that the product of aldol addition between two acetaldehyde molecules, 3-hydroxybutanal, is prone to water elimination leading to formation of crotonaldehyde, which is then able to covalently crosslink catalytic Lys167 and Cys47 in the active site (Dick et al. 2016a). This observation led Bramski et al. to prepare seven *E. coli* DERA variants in which Cys47 was mutated to Methione, Serine, Threonine, Leucine, Isoleucine, Alanine, or Glycine (Bramski et al. 2017). The stability of each variant against acetaldehyde and crotonaldehyde was tested together with the specific activity in retroaldol reaction by using DR5P as substrate. *E. coli* DERA Cys47Leu mutation was found to be the most effective, having 30-fold increased half-life for crotonaldehyde and 15-fold for acetaldehyde. However, specific activity for DR5P retroaldol reaction decreased by 23-fold. Haridas and coworkers (Haridas et al. 2020) have also recently improved the acetaldehyde tolerance of *Pectobacterium atrosepticum* DERA by creating a Cys49Met mutation. It is worth noting that *P. atrosepticum* DERA has an optimum pH in the alkaline range between 8.0 and 9.0, whereas *E. coli* DERA which shows a maximal activity at more neutral pH range (between pH 6–8). Similar to the *E. coli* DERA, this mutation led to significant improvements in the acetaldehyde tolerance of *P. atrosepticum* DERA.

Kim et al. (2020) have utilized DERA in synthesis of *(R)-*1,3-butanediol (1,3-BDO) which can be used as a precursor for different kinds of polymers and biologically active compounds (Kim et al. 2020). In this scheme, DERA catalyzes coupling of two acetaldehydes to form 3-hydroxybutanal which is then reduced to 1,3-BDO by aldo-keto reductase. Altogether 20 DERA aldolases from different organisms were initially screened on the basis of 1,3-BDO production. DERA BH1352 from *Bacillus halodurans* was chosen for further studies and active site mutations based on the solved crystal structure were created. This was done by targeting hydrophobic residues near the catalytic Lys155. Mutants were tested for retroaldol reaction by using DR5P as a substrate and for aldol reaction by following formation of 1,3-BDO. In retroaldol reaction, Phe160Tyr mutation was 23% more active compared to *B. halodurans* DERA wild-type enzyme. The same mutation increased 1,3-BDO synthesis almost 3-fold. Phe160 is not conserved among DERAs. It corresponds to Lys172 in *E. coli* DERA being one of the phosphate-binding residues. Another *B. halodurans* DERA variant with double mutation Phe160Tyr/Met173Ile was even more effective. Here the Met173 corresponds to Met185 in *E. coli* DERA, where it has been also been shown to contribute to the sequential aldol condensation (Jennewein et al. 2006).

## Homologous grafting

Bisterfeld et al. (2016) introduced a term “homologous grafting” for their protein engineering approach which was used to engineer the stereoselectivity of *E. coli* DERA (Bisterfeld et al. 2016). They utilized the structural information from two other *E. coli* aldolases, namely pyruvate-dependent 2-keto-3-deoxy-6-phosphogluconate aldolase (KDPG) and 2-keto-3-deoxy-6-phosphogalactonate aldolase (KDPGal), to design the mutations. All three aldolases are TIM barrel proteins and Class I aldolases that utilize glyceraldehyde-3-phosphate (G3P) as the electrophile. The two pyruvate-dependent homologous aldolases, which accept otherwise similar substrates but have different stereospecificities towards one stereogenic carbon, were analyzed using geometrics and molecular dynamics (MD) simulations as well as using phylogenetic analysis. This led to the identification of three amino acid positions which could affect stereoselectivity. The corresponding positions in *E. coli* DERA (Thr18, Leu20, and Ala203) were then studied by saturation mutagenesis in two rounds, in terms of enantiomeric excess of aldol reaction using acetaldehyde and propanal as substrates. Mutations at Thr18 and Leu20 had the largest effect on stereoselectivity without significant decrease of enzymatic activity. It was concluded that Thr18Ser was the most important residue contributing to stereoselectivity (Bisterfeld et al. 2016). In the complex structures of wild-type *E. coli* DERA (1JCL), the methyl group of Thr18 packs against the targeted stereogenic carbon which could explain the decrease in stereoselectivity (Fig. 3B).

## Directed evolution of DERA enzymes

In order to improve the applicability of DERA for industrial synthesis of (3R,5S)-6-chloro-2,4,6-trideoxyhexapyranoside, a precursor for atorvastatin drug, Jennewein et al. utilized various protein engineering strategies (Jennewein et al. 2006). The desired reaction couples one chloroacetaldehyde and two acetaldehyde molecules to form a cyclic hexapyranose which can be further oxidized to the derivative of mevinic acid, a precursor for manufacturing of the statin drugs. Directed evolution was used to improve the resistance of *E. coli* DERA as well as the catalytic efficiency towards chloroacetaldehyde. The stability and activity screenings were carried out initially separately. Stability screening involved three rounds of mutagenesis and screening, where first a library with over 20,000 random mutants having 2–5 amino acid mutations per clone was created. After screening the cell-free extracts where DR5P retroaldol reaction was assayed in the presence of chloroacetaldehyde, two rounds of recombination of the best mutants were carried out. In each case, 3000 clones were screened in a similar fashion as in the 1st round. This led finally to identification of four *E. coli* DERA variants (DERA^Var2^, DERA^Var3^, DERA^Var4^, and DERA^Var5^) having substantially improved tolerance to chloroacetaldehyde. Here, three amino acid positions could be identified that affected the stability improvements. The authors discuss the rational of these mutations based on the solved 3D structure of *E. coli* DERA: the residues Phe200, Ile166, and Met185 together seem to form a small hydrophobic cluster close to the peptide backbone of the active site Lys167 and Lys201. The authors also noticed that Lysine residues on *E. coli* DERA solvent-exposed surface were modified upon incubation with chloroacetaldehyde. Utilization of site-specific mutagenesis of individual Lysine residues did not, however, lead to a detectable improvement in chloroacetaldehyde tolerance of the tested variant DERA variants.

After the chloroacetaldehyde resistance work, Jennewein and coworkers (Jennewein et al. 2006) used the previously created random mutant library to screen for improved activity in an assay set-up where chloroacetaldehyde and acetaldehyde were used as substrates. This resulted in seven *E. coli* DERA variants showing at least 3-fold improvement in product formation compared to the wild-type *E. coli* DERA (designated DERA ^Var6–Var12^). The best activity improvement with regard to (3R,5S)-6-chloro-2,4,6-trideoxyhexapyranoside formation was obtained with DERA^Var9^ (having single Phe200Ile substitution). This substitution led to a reduction in the *K*_m_ value (from 55 ± 5 mM to 24 ± 4 mM) for chloroacetaldehyde. The mutated residue is located in the β-strand of the β-barrel (Fig. 3B). Although the side chain is near the active site, it is pointing out from the active site and does not directly interact with substrate. This mutation may cause small alterations in the β-barrel structure. In addition, the sequencing of the variants revealed again the importance of the mutations at position Met185 (DERA^Var11^, Met185Thr), similarly to the stability mutants. In the end, mutations obtained in the stability and activity screens were combined. Here in particular, the mutation leading to the Phe200Ile substitution was combined with mutations affecting the C-terminus, either resulting in the C-terminal Tyr259 deletion, or its substitution (Tyr259Lys) in combination with the addition of 9 additional amino acid residues, respectively. These studies showed synergistic action for the mutations, leading to further improvements of DERA as a catalyst for the stereoselective synthesis of (3R,5S)-6-chloro-2,4,6-trideoxyhexapyranoside. The obtained variants DERAVar^13^ (Phe200Ile/ΔTyr259) and DERA^Var14^ (Phe200Ile/Ser258Thr/Tyr259Thr + C-terminal extension KTQLSCTKW) showed at 500 mM chloroacetaldehyde and 1 M acetaldehyde concentration, 61% and 70% conversion, respectively, to (3*R,*5*S*)-6-chloro-2,4,6-trideoxyhexapyranoside after 8 h. In contrast with wild-type DERA, only a minor amount of product was obtained under similar conditions.

Jiao et al. (2017) have used random mutagenesis to improve the catalytic efficiency of *Lactobacillus brevis* DERA in sequential aldol reaction in synthesis of (3R,5S)-6-chloro-2,4,6-trideoxyhexapyranoside (Jiao et al. 2017). Altogether 2000 clones were tested by assaying reaction mixture containing chloroacetaldehyde and acetaldehyde. The reaction mixture contained also *Lodderomyces elongisporus* aldehyde dehydrogenase which oxidized the aldol product to the corresponding lactone. Two mutants, Thr29Val and Thr29Leu, showed improved activities. Thr29 (corresponding to Leu32 in *E. coli* DERA, Fig. 3B) is located in an alpha helix, and the side chain is located on the interface between the β-barrel and the α-helix. Its substitution may thus cause only a minor change in the shape of the active site. Additional mutations were subsequently searched for the Thr29Leu variant by targeting the loops of the active site. Although higher activities were found, for example, for the mutant Thr29Leu/Phe163Tyr, the original single mutation Thr29Leu produced a higher yield for the product in a shorter time, probably because of its better tolerance at high aldehyde concentrations.

## Machine learning (ML)–guided protein engineering

**Fig. 4:**
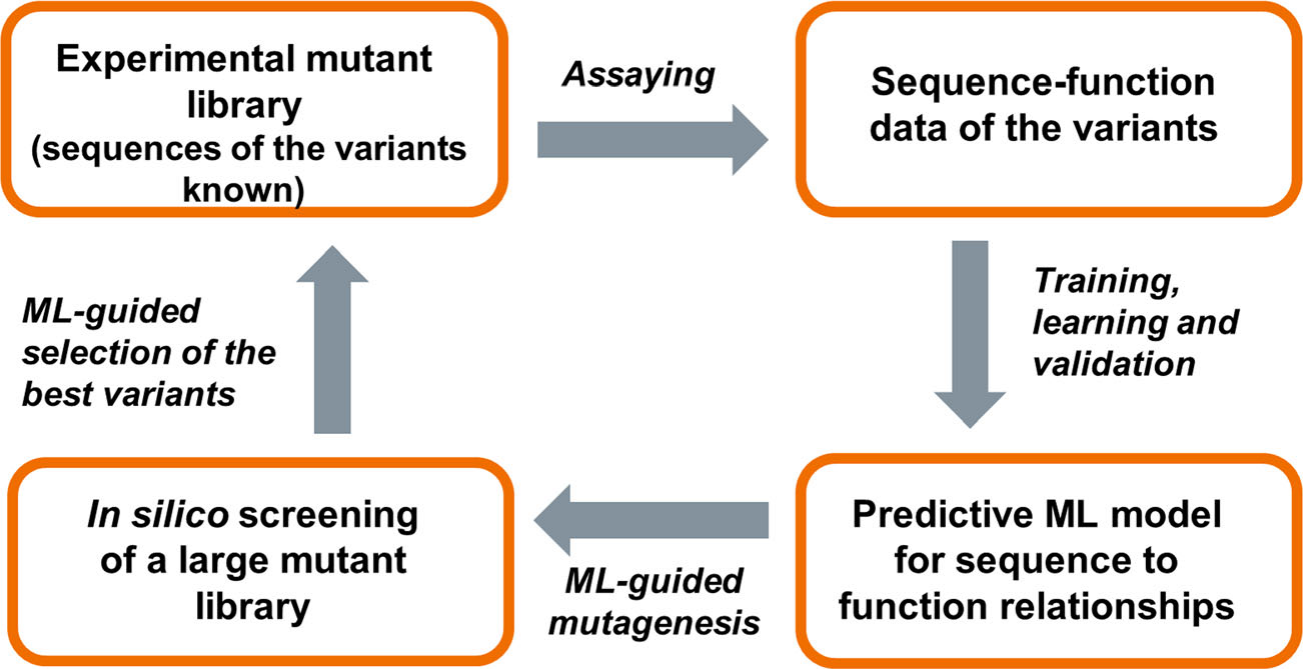
A schematic picture how machine learning (ML) methods can be applied to accelerate protein engineering work. ML models can be trained from a relatively limited set of experimental variant data to predict how amino acid sequence maps to function without requiring information about the 3D protein structure. The ML models can be used to predict the properties of variants not experimentally evaluated and to propose a new, limited set of experimental variants to be screened in the next evolution round in order to improve the protein properties

Machine learning (ML) models can predict how an amino acid sequence correlates to protein function in a data-driven manner, independently of the 3D structure. These methods have a potential to accelerate directed evolution by reducing the number of variants and evolution cycles, as they can learn from the properties of characterized enzyme variants and use that information to select sequences that are likely to exhibit improved properties (Fig. 4). It is also worth noting that the ML-based methods are not iterative as in classical directed evolution, but they can also generate new, previously unseen variants with promising properties. There are several recent review articles on how machine learning can be used to guide the directed evolution (Yang et al. 2019; Mazurenko et al. 2020; Wittmann et al. 2021).

Voutilainen et al. have recently used three rounds of mutagenesis combined with a ML method to create variants of *E. coli* DERA having improved catalytic activity for acetaldehyde aldol reaction, combined with clearly reduced activity for DR5P and DR retroaldol reaction (Voutilainen et al. 2020). At first round, 69 single DERA mutants were created targeting 24 amino acid positions selected mainly by using 3D structures, literature data, and bioinformatics tools, such as Hotspot Wizard (Bendl et al. 2016). The catalytic properties of the mutants were tested in three different reactions: to retroaldol direction by using DR5P and DR as substrates and aldol direction by using acetaldehyde as a single substrate. Five mutants (Gly204Ala, Ser239Glu, Leu17Gly, Gly171Ala, and Gly171Ser) showed increased activity for the aldol reaction, and reduced activity for the retroaldol reaction towards DR5P. In the second round, 62 variants containing two or three amino acid mutations were tested. The additional mutant positions were selected either manually by utilizing data from the previous round or by using saturation mutagenesis on selected positions. A number of variants were found showing reduced retroaldol activity towards DR5P and DR as well as improved aldol activity towards acetaldehyde.

The results from the first and second round contained biochemical characterization and sequence data for 131 *E. coli* DERA variants, which was used to train the ML model to automatically predict all three substrate specificities (on DR5P, DR, and acetaldehyde) (Voutilainen et al. 2020). Altogether 48,000 new *E. coli* DERA variants having 1–3 amino acid mutations were then created and screened in silico. From 50 top DERA variants having highest aldol activity on acetaldehyde and low retroaldol activity towards DR5P, 18 *E. coli* DERA mutants were finally expressed, purified, and tested. All had minimal activity towards DR5P and DR but improved activity in acetaldehyde aldol reaction, thus demonstrating the power of computer-aided tools in protein engineering. Five best DERA variants had more than twofold improved activity towards acetaldehyde: Cys47Val/Gly204Ala/Ser239Asp, Asn21Ser/Cys47Glu/Gly204Ala, Cys47Val/Gly204Ala/Ser239Glu, Cys47Val/Gly204Ala, and Asn21Lys/Cys47Val/Gly204Ala (Table 2). When considering all the mutant data created in this work, many beneficial mutant positions are in the substrate-binding site where the phosphate group of DR5P binds (namely Gly171, Gly204, Ser238, Ser239, Asn21). Some of these mutations were shown to cause conformational changes in the substrate-binding site, narrowing the entrance. Creating negative charge near the phosphate-binding site, e.g. through Ser239Asp mutation, also likely contributed to the decreased phosphate binding. All the best DERA variants included also mutation at Cys47, which has been shown also in other studies to be a beneficial target for substitutions, probably due to decreased covalent inhibitory adduct formation with aldehydes (Voutilainen et al. 2020). The DERA from a hyperthermophilic *A. pernix* has naturally a Valine residue at this position.

## Other means to improve DERA properties for application purposes

Enzyme immobilization on solid support is often used in applications, as it facilitates separation, may be reused, and permits continuous operation. The drawback is that the immobilization may lower the specific activity of the enzyme, e.g. due to steric hindrances. However, there are also reports that through immobilization improved enzyme properties can be obtained. Thus, immobilization may act in some cases as an alternative or additional approach to protein engineering. As an example, *E. coli* DERA has been immobilized on multiwalled carbon nanotubes, and the catalytic performance was evaluated in the self-aldol reaction of acetaldehyde and using chloroacetaldehyde. DERA in immobilized form was shown to exhibit tolerance to higher acetaldehyde concentration and the enzyme activity was maintained after five cycles (Subrizi et al. 2014). Stable and efficient enzyme immobilization technique could also be established by immobilizing *E. coli* DERA on glutaraldehyde-(3-aminopropyl)triethoxysilane nano-magnet material (GA-APTES-NSM). Over 75% of the applied enzyme was immobilized and an increase in tolerance to higher substrate concentrations was observed. Immobilized enzyme also exhibited enhanced thermal stability (Fei et al. 2014). In addition to nanomaterials as immobilization matrix, *E. coli* DERA has also been immobilized on thin polymeric films (Zhang et al. 2018; 2019). The conjugation of the synthetic polymer to DERA led to increased acetaldehyde tolerance of the enzyme (Zhang 2018). In addition, crosslinking of the DERA-polymer onto a polymeric membrane support allowed the active membrane to be tested in a continuous-flow mode process. Low amounts of aldol product could be observed using hexanal and acetaldehyde as substrates (Zhang et al. 2019). A continuous-flow process was also developed by Grabner et al. for the synthesis of statin side-chain precursor. In this process, freeze-dried whole *E. coli* cells expressing the C47M DERA mutant were immobilized using alginate-luffa matrix (Grabner et al. 2020).

Besides immobilization, the use of whole cell biocatalysts is an attractive alternative in industrial processes, not only due to the low production cost, but also as a tool to stabilize the enzymes (Lin and Tao 2017). *E. coli* cells overexpressing *E. coli* DERA have been used as a whole cell biocatalyst to produce, e.g., lactonized side-chain intermediates used in statin synthesis (Ošlaj et al. 2013). When the bacterial cells from a fed-batch, high-density bioreactor cultivation were used, the DERA-catalyzed sequential aldol addition reaction of acetaldehyde was shown to be highly productive and cost-efficient with products having high enantiomeric purities (Ošlaj et al. 2013). In another study by Feron et al. (2007) *E. coli* cells overexpressing *E. coli* DERA were used to produce 4-hydroxybenzylidene acetone, a precursor of raspberry ketone which is the key component of raspberry flavour. DERA catalyzed an aldol reaction between 4-hydroxybenzaldehyde and acetone to produce 4-hydroxybenzylidene acetone. An advantage of this process was that the bacterium also catalyzed the beta-elimination of water from the direct product of the aldol reaction, thus preventing further reactions to take place (Feron et al. 2007). In a third example, higher substrate tolerance and increased product yield were achieved when *E. coli* cells overexpressing a mutated *Klebsiella pneumoniae* DERA were used for the biosynthesis of D-2-deoxyribose from D-glyceraldehyde and acetaldehyde as compared to in vitro enzymatic synthesis (Li et al. 2015). As also mentioned earlier, the mutated KDERA^K12^ (*K. pneumoniae* DERA Ser238Asp/Phe200Ile/ΔTyr259) enzyme showed in vitro 3 times higher specific activity and 1.5-fold increased substrate tolerance as compared to the wild-type enzyme. By using whole cell biocatalysis approach, the substrate (aldehyde) tolerance of KDERA^K12^ could be further improved (from 0.5 M to 3 M) (Li et al. 2015).

## Multistep enzyme cascades in vitro and in vivo

Besides single-enzyme catalyzed reactions, DERA aldolases have also been applied in vitro and in vivo in sequential biocatalytic reactions. Multi-enzyme cascades avoid the waste generated by purification of intermediates and they also allow reactions to be linked together to overcome an unfavourable equilibrium, or avoid the accumulation of unstable or inhibitory intermediates. Furthermore, in the case of microbial pathways (in vivo enzyme cascades), diverse new compounds can be synthetized directly from renewable sources.

As a proof-of-concept, DERA-based pathway has been engineered in *E. coli* cells to produce 1,3-BDO from glucose, involving three heterologous enzymes: pyruvate decarboxylase (PDC, producing acetaldehyde from pyruvate), DERA (catalyzing aldol addition of two acetaldehyde molecules to 3-hydroxybutanal), and aldo-keto reductase (AKR), which reduces 3-hydroxybutanal (3-HB) to 1,3-BDO (Nemr et al. 2018). The product titer and yield were further improved using metabolic engineering approaches, which included reducing major by-products and increasing pathway flux through DERA to reduce accumulation of toxic acetaldehyde, as well as implementing a two-stage fermentation process. As a follow-up of this work, Kim et al. (2020) screened 20 microbial DERAs for the most potent enzyme on acetaldehyde addition reaction and identified *Bacillus halodurans* DERA as a potent aldolase for production of 1,3-BDO from acetaldehyde. As also described above, structure-based site-directed mutagenesis was further used to create *B. halodurans* DERA variants with higher activity in the production of 1,3-BDO. The replacement of the wild-type DERA by the Phe160Tyr or Phe160Tyr/Met173Ile variants in *E. coli* cells expressing the DERA+AKR pathway increased the production of 1,3-BDO from glucose five and six times, respectively.

Several research groups have utilized DERA enzyme in combination with other enzymes in in vitro synthesis studies. Horinouchi et al. (2006) coupled *Klebsiella pneumoniae* DERA as a last step in synthesis of DRP from D-glucose by utilizing first the glycolytic enzymes of baker’s yeast (*S. cerevisiae*) to produce fructose 1,6-diphosphate that was then used by the DERA-expressing *E. coli* cells*.* Honda and coworkers (Honda et al. 2010) used DERA and five other thermophilic enzymes from *T. thermophilus* in a multi-enzyme cascade to produce DRP from fructose with a high yield. Furthermore, van Herk et al. (2009) tested a two-enzyme cascade reaction consisting of the Val78Leu mutant of a non-specific acid phosphatase and DERA to produce DRP from DL-glyceraldehyde. As a last example, a multi-enzyme cascade has recently been used in vitro to construct investigational Islatravir drug for HIV treatment from simple building blocks in a collaborative effort by Merck and Codexis (Huffman et al. 2019). The overall biocatalytic synthesis route required clearly less steps than the previously reported routes. For this cascade, the *Shewanella halifaxensis* DERA was chosen based on its high activity, stereoselectivity (> 99% de), and kinetic selectivity favouring reaction with *(R)*-enantiomer of the aldehyde. Two rounds of directed evolution were carried out to obtain a *S. halifaxensis* DERA variant that retained high activity at an acetaldehyde concentration > 400 mM. This variant contained altogether 11 amino acid changes (Ser2Cys; Gln10Arg; Cys47Val; Asp66Leu; Ala71Val; Ala145Lys; Leu156Ile; Met184Ile; Val203Ile; Ser235Thr; Ser236Asp), including also the mutation at the Cys47, shown also by others to affect the acetaldehyde inhibition.

## Conclusions and future perspectives

DERA is a potential industrial biocatalyst for C–C bond formation reactions. There are already many examples where DERA has been utilized in stereoselective enzyme-assisted synthesis of commodity chemicals, flavours, and building blocks for pharmaceutical drug molecules. Different protein engineering approaches have also been successfully used in improving the substrate specificity, catalytic efficiency, and lifetime towards native and non-native substrates and reactions. These studies demonstrate that the TIM barrel fold of DERA aldolases offers a good starting point to develop novel biocatalysts. Many of the studies have been carried out using *E. coli* DERA; however, there are a multitude of other DERA enzymes that would also offer a good starting point for protein engineering and usage as a biocatalyst in various applications. As there is a requirement for moving towards more sustainable future, we should be able to create faster and more efficient biocatalysts to produce completely new type of products. The successful design of optimal biocatalysts requires not only use of advanced protein engineering strategies, but it would greatly benefit of having more high-accuracy biochemical data available, particularly involving data on enzyme kinetics and product analysis. There is currently a huge amount of gene sequencing data available, but the biochemical characterization of enzymes is lacking behind, thus hindering the realization of the full potential of data-driven computational tools.

Concerning future perspectives, the most extreme case of protein engineering is de novo protein design, where completely non-natural proteins are designed “from scratch” based purely on biophysical principles (Jiang et al. 2008; Huang et al. 2016). Here the challenge is to identify a protein sequence that will fold into a desired structure and function. Protein modelling software, such as Rosetta (Rohl et al. 2004; Alford et al. 2017), is an indispensable tool for screening in silico sequence candidates for a desired fold. The kinetic parameters of de novo designed enzymes are still clearly lower than those of naturally occurring ones, but here again directed evolution can be utilized to improve the properties. As an example of these types of efforts, *E. coli* DERA TIM barrel scaffold has been utilized to create an artificial enzyme that can carry out an unnatural reaction, named as Kemp elimination (Khersonsky et al. 2011). Furthermore, the recent development in artificial intelligence (AI)–based approaches, such as the AlphaFold (Callaway 2020) that can be used for determining a protein’s 3D structure from its amino acid sequence, is expected to further speed up the progress.

## Data Availability

Not applicable.
